# Treatment patterns and their outcomes of acute aortic intramural hematoma in real world: multicenter registry for aortic intramural hematoma

**DOI:** 10.1186/1471-2261-14-103

**Published:** 2014-08-19

**Authors:** Yoon-Jung Choi, Jang-Won Son, Sang-Hee Lee, Ung Kim, Dong-Gu Shin, Young-Jo Kim, Seung-Ho Hur, Chang-Wook Nam, Yun-Kyeong Cho, Bong-Ryul Lee, Byung-Chun Jeong, Jin-Bae Lee, Jae-Kean Ryu, Hun-Sik Park, Jang-Hoon Lee, Se-Yong Jang, Jong-Seon Park

**Affiliations:** 1 Division of Cardiology, Department of Internal Medicine, Yeungnam University Medical Center, 170, Hyeonchung-ro, Nam-gu, Daegu 705-717, Republic of Korea; 2 Division of Cardiology, Department of Internal Medicine, Keimyung University Dongsan Medical Center, 56, Dalsung-ro, Jung-gu, Daegu 700-712, Republic of Korea; 3 Division of Cardiology, Department of Internal Medicine, Daegu Fatima Hospital, 99, Ayang-ro, Dong-gu, Daegu 701-724, Republic of Korea; 4 Division of Cardiology, Department of Internal Medicine, Daegu Catholic University Medical Center, 17, Duryugongwon-ro, Nam-gu, Daegu 705-718, Republic of Korea; 5 Division of Cardiology, Department of Internal Medicine, Kyungpook National University Hospital, 130, Dongduk-ro, Jung-gu, Daegu 700-721, Republic of Korea

**Keywords:** Intramural hematoma of the aorta, Survival rates, Treatment

## Abstract

**Background:**

Intramural hematoma of the aorta (IMH), a variant of classic aortic dissection, shows very dynamic process in the early phase. The aim of this study is to evaluate clinical outcomes of patients with acute aortic IMH from real world registry data.

**Methods:**

We analyzed 165 consecutive patients with acute IMH from five medical centers in Korea. All patients were divided into two groups; type A (n = 61, 37.0%) and type B (n = 104, 63.0%) according to the Stanford classification. Clinical outcomes and morphological evolution by CT were analyzed for 2 years.

**Results:**

Most of the patients (77.0% of type A and 99.0% of type B, *P* < 0.001) were treated medically during their initial hospitalization. There were no significant differences in in-hospital mortality (4.9% vs. 2.9%, *P* = 0.671) and 2-year mortality (13.1% vs. 11.5%, *P* = 0.765) between two groups. During the 2-year follow up period, progression to aortic dissection (18.0% vs. 6.7%, *P* = 0.037) and surgical treatment (29.5% vs. 2.9%, *P* < 0.001) were higher in type A. For the type A patients, there were no significant difference in in-hospital mortality (7.1% of surgery vs. 4.3% of medical, *P* = 0.428) and 2-year mortality (7.1% of surgery vs. 14.9% of medical, *P* = 0.450) in terms of initial treatment strategy.

**Conclusion:**

For real world practice in Korea, most of IMH patients were treated medically at presentation and showed favorable outcomes. Thus, even in type A acute IMH, early medical treatment with alternative surgical conversion for selected, complicated cases would be a favorable treatment option.

## Background

Aortic intramural hematoma (IMH) is an important acute aortic syndrome that presents symptoms similar to those of classic aortic dissection. Recent advances in imaging techniques have significantly improved the diagnosis and heightened the clinical understanding of IMH, which accounts for a frequency of 10% to 30% of all acute aortic syndromes [1-3]. IMH is different from aortic dissection due to its absence of continuous direct flow communication through intimal tear; It shows very dynamic process in the early phase and the natural history of IMH is not clearly known yet.

IMH is classified as either involving (type A) or not involving (type B) the ascending aorta. The site of IMH is an important parameter to determine prognosis. Patients with type B IMH, like those with type B aortic dissection, are usually treated medically [4]. However, management of patients with type A IMH is controversial. Some reports have recommended early surgery for patients with type A IMH because of their poor prognosis with medical treatment [5-7]. On the other hand, recent studies have shown favorable results with medical therapy in some type A IMH cases [8,9]. Therefore, we retrospectively analyzed imaging studies, treatment strategies and clinical outcomes of patients with acute IMH from real world registry data.

## Method

### Study patients

From January 2000 through December 2010, a total of 165 patients with acute IMH were admitted to five medical centers. IMH was defined as a circular or crescent shape, extensive aortic wall hematoma with no evidence of intimal flap or false lumen noted by contrast computed tomographic (CT) scan. The patients were divided into two groups according to the aortic segment afflicted with IMH, whether it was on the ascending thoracic aorta and/or aortic arch (type A, n = 61) or the descending thoracic aorta (type B, n = 104). Clinical features, hospital mortality, and long-term survivals were compared between patients with type A and type B IMH for 2 years.

### Data collection and analysis

Chart review was performed, and data were collected with a standardized format that included information on patient demographics, medical history, clinical presentation, physical findings, results of imaging studies, details of medical and surgical treatment, and outcome. Follow-up data were collected by direct telephone interviews and detailed review of all medical records. The cause and date of any death were confirmed by information gathered from the National Population Registry of the Korean National Statistical Office, together with a review of all available clinical records at the time of death. This retrospective study was approved by the Institutional review board of Yeungnam University (IRB No. YUH-13-0362-B3). The need for informed consent was waived by the board.

### Statistical analysis

Categorical variables are described as number and percent and compared with the chi-squared test, or Fisher exact test as appropriate. Continuous variables are described as mean ± SD. Survival analysis was performed by Kaplan-Meier analysis, and differences in survival between the groups were examined with the log-rank test. To determined predictors for progression of ascending IMH and mortality throughout follow-up period, Cox proportional hazards model was used to estimate the risk of the potential variables. *P* value < 0.05 (two-sided) was considered statistically significant. Data analyses were performed with SPSS software (version 18.0; SPSS, Inc, Chicago, Ill).

## Results

**Figure 1:**
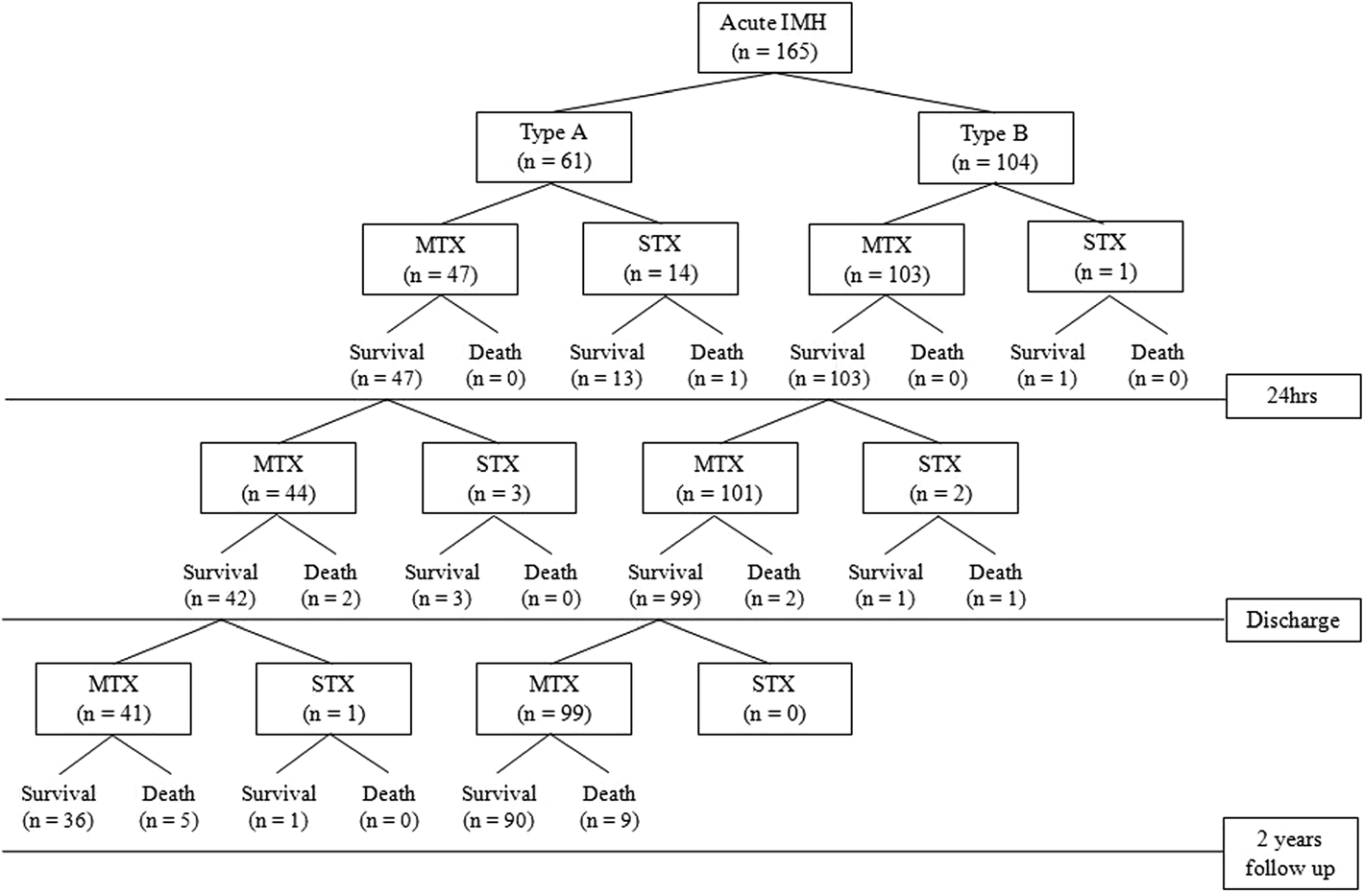
**Diagram of enrolled patients and clinical outcomes.** MTX, medical treatment; STX, surgical treatment.

Table 1 summarizes the clinical features of both groups. Mean age (68 ± 10 vs. 67 ± 12, *P* = 0.521), the prevalence of diabetes (6.6% vs. 6.7%, *P* = 0.966), hypertension (60.7% vs. 50.0%. *P* = 0.185), dyslipidemia (36.4% vs. 31.6%, *P* = 0.688), current smoking status (32.8% vs. 28.8%, *P* = 0.595) and time from onset to admission (10.6 ± 16.2 vs. 7.9 ± 11.3, *P* = 0.251) did not show any difference between the two groups. Male gender was more frequently observed in the type B group (37.7% vs. 58.7%, *P* = 0.009). Chest pain was the most prevalent symptom in both groups. Abdominal pain and back pain followed that. Initial systolic blood pressure was higher in the type B group (138 ± 36 vs. 150 ± 36, *P* = 0.042). Diastolic blood pressure (84 ± 21 vs. 86 ± 19, *P* = 0.522) and heart rate (77 ± 18 vs. 77 ± 23, *P* = 0.997) were no statistically significantly different between the two groups. The laboratory findings were also similar in the two groups.Selected treatment modalities and clinical outcomes are summarized in Figure 1. In the type A group, 23% (14/61) underwent emergent surgery, and medical treatment was selected for the remaining 47 patients. Among patients who were treated medically at initial presentation, 3 patients underwent surgery during the hospitalization because of the development of hemopericardium, aggravation of pleural effusion and dye leakage detected from follow up CT. After discharge, only one patient received surgery because of the development of aortic dissection. In the type B group, one patient underwent emergent surgery because suspicious penetrating ulcer was seen in CT. Additional 2 patients underwent timely surgery after enlarged aortic diameter (up to 63 mm) and extravasation of dye were seen from follow up CT examination. Most patients with type B (101/104, 97.1%) IMH received medical treatment only. A total of 21 patients who were underwent surgery during 2 years, 20 cases had aorta graft replacement and 1case had hybrid EVAR (endovascular aneurysm repair).

**Table 1:** Clinical characteristics of the patients, by location of aortic intramural hematoma

	**Type A (n = 61)**	**Type B (n = 104)**	***P *** **value**
Age, yrs	68 ± 10	67 ± 12	0.521
Sex, male	23 (37.7%)	61 (58.7%)	0.009
Diabetes	4 (6.6%)	7 (6.7%)	0.966
Hypertension	37 (60.7%)	52 (50.0%)	0.185
Dyslipidemia	16 (36.4%)	24 (31.6%)	0.688
Current smoking	20 (32.8%)	30 (28.8%)	0.595
Time from onset to admission (hrs)	10.6 ± 16.2	7.9 ± 11.3	0.251
**Initial symptoms**			0.372
Chest pain	31 (50.8%)	58 (55.8%)	0.538
Abdominal pain	11 (18.0%)	21 (20.2%)	0.735
Back pain	10 (16.4%)	19 (18.3%)	0.760
Loss of consciousness	2 (3.3%)	3 (2.9%)	1.000
Dyspnea	4 (6.6%)	1 (1.0%)	0.159
Others	3 (4.9%)	2 (1.2%)	0.360
**Hemodynamic findings**			
Systolic BP, mmHg	138 ± 36	150 ± 36	0.042
Diastolic BP, mmHg	84 ± 21	86 ± 19	0.522
Heart rate, BPM	77 ± 18	77 ± 23	0.997
**Laboratory findings**			
Total cholesterol (mg/dL)	185 ± 53	180 ± 46	0.550
C-reactive protein (mg/dL)	3.2 ± 4.3	4.7 ± 8.3	0.236
Creatinine (mg/dL)	1.20 ± 1.50	0.97 ± 0.34	0.279

**Figure 2:**
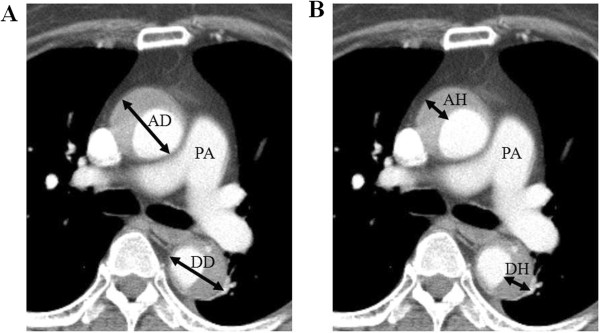
**Measurement methods of intramural hematoma of the aorta.** Maximal aortic diameter **(A)** and maximal hematoma thickness **(B)** were measured at the level of pulmonary artery bifurcation. AD, Maximal ascending aortic diameter; AH, Maximal ascending hematoma thickness; DD, Maximal descending aortic diameter; DH, Maximal descending hematoma thickness; PA, Pulmonary artery.

The results of CT were demonstrated in Table 2 according to the time of examinations. Initial, in-hospital follow up, outpatient department follow up CT results were seen. With the use of computerized planimetry, measurements were taken on the basis of the accompanying calibrated scales in the contrast-enhanced CT images. Maximal aortic diameter and maximal hematoma thickness were measured at the level of pulmonary artery bifurcation (Figure 2). Maximal ascending aortic diameter in initial CT images was significantly greater in the type A group (47.8 ± 8.2 mm vs. 42.1 ± 5.7 mm, *P* < 0.001). Maximal descending aortic diameter was greater in the type B group, but there was no significant difference (37.7 ± 7.4 mm vs. 40.0 ± 8.1 mm, *P* = 0.084). Maximal descending hematoma thickness in type A was 10.8 ± 6.8 mm and maximal descending hematoma thickness in type B was 11.6 ± 5.1 mm. Pericardial effusion was common in the type A group (32.8% vs. 3.9%, *P* < 0.001). In-hospital follow up CT was performed in 78.8% of patients (mean duration 10 ± 7 days). Among them, 59.6% of type A and 41.0% of type B patients showed a decreasing trend of hematoma thickness. There were no significant differences of hematoma thickness (less than 2 mm) compared to initial CT in the 34.0% of type A and 50.0% of type B patients. Progression to aortic dissection was common in type A patients (12.8% vs. 3.6%, *P* = 0.069). Outpatient department follow up CT was performed only in 58.2%. However, information from the National Population Registry of the Korean National Statistical Office and direct telephone interview confirmed that other patients were asymptomatic and in stable condition except the person who died. To determine the independent predictors for progression of type A IMH throughout the follow-up period, Cox regression analysis was performed. There was no significant predictor of the progression of IMH. However, aortic hematoma thickness greater than 10 mm was associated with increased mortality (odds ratio, 9.23; 95% Confidence interval (CI), 1.30 to 65.4; *P* = 0.026) (Table 3).

**Table 2:** The results of CT examinations

	**Type A (n = 61)**	**Type B (n = 104)**	***P *** **value**
**Initial computed tomography**			
Maximal ascending aortic diameter, mm	47.8 ± 8.2	42.1 ± 5.7	< 0.001
Maximal descending aortic diameter, mm	37.7 ± 7.4	40.0 ± 8.1	0.084
Maximal ascending hematoma thickness, mm	10.8 ± 6.8	-	< 0.001
Maximal descending hematoma thickness, mm	9.9 ± 5.0	11.6 ± 5.1	0.042
Pleural effusion	14 (23.0%)	22 (21.4%)	0.812
Pericardial effusion	20 (32.8%)	4 (3.9%)	< 0.001
**In-hospital follow up CT**			
Follow-up CT duration, days	10 ± 7	9 ± 7	0.483
Follow-up CT study	47 (77.0%)	84 (80.8%)	0.568
Decreased hematoma	28 (59.6%)	34 (41.0%)	0.041
Increased hematoma	3 (6.4%)	7 (8.4%)	0.480
Progression to aortic dissection	6 (12.8%)	3 (3.6%)	0.069
No change*	16 (34.0%)	42 (50.0%)	0.068
**Outpatient Department follow up CT**			
Follow-up CT duration, days	452 ± 724	318 ± 453	0.267
Follow-up CT study	38 (62.3%)	58 (55.8%)	0.412
Decreased hematoma	29 (78.4%)	48 (82.8%)	0.401
Increased hematoma	3 (7.9%)	4 (6.9%)	1.000
Progression to aortic dissection	5 (13.2%)	5 (8.6%)	0.510
No change*	6 (15.8%)	6 (10.3%)	0.430

*No change; Change of hematoma thickness is less than 2 mm compared to initial CT.

**Table 3:** Predictors of 2-year mortality by Cox regression analysis

	**Odds ratio**	**95% confidence interval**	***P *** **value**
Age > 70 years	1.64	0.23 - 11.78	0.623
Diabetes	0	0	0.987
Hypertension	2.81	0.42 - 18.61	0.285
Dyslipidemia	0.26	0.02 - 2.99	0.278
Time from onset to admission > 24 hrs	1.67	0.25 - 11.09	0.600
Initial systolic BP < 90 mmHg	3.00	0.04 - 202.96	0.609
Pleural effusion at CT	0.38	0.02 - 0.68	0.509
Pericardial effusion at CT	3.51	0.18 - 67.84	0.405
Maximal ascending aortic diameter > 55 mm	1.43	0.17 - 11.69	0.740
Maximal ascending hematoma thickness > 10 mm	9.23	1.30 - 65.4	0.026

**Figure 3:**
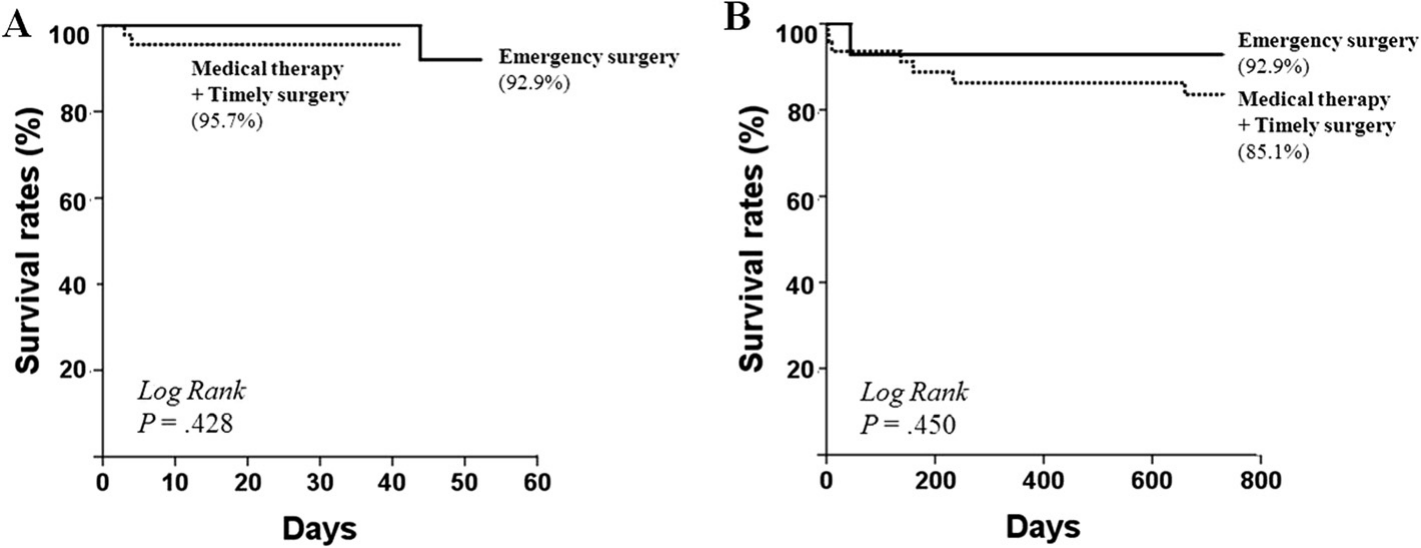
**Survival rates of type A acute intramural hematoma according to the initial treatment strategy. (A)** In-hospital survival rates were 92.9% who received emergency surgery vs. 95.7% who received medical therapy and timely surgery in selected patients (*P* = 0.428). **(B)** During 2-year follow up, Survival rates were 92.9% who received emergency surgery vs. 85.1% who received medical therapy and timely surgery in selected patients (*P* = 0.450).

Table 4 shows treatment strategy and clinical outcomes according to location of IMH. Patients receiving emergent surgery had been hospitalized longer than those receiving medical therapy in both type A (29 ± 14 vs. 16 ± 8, *P* = 0.004) and type B (26 vs. 17 ± 13, *P* = 0.492) groups. During the 2-year follow up period, progression to aortic dissection (18.0% vs. 6.7%, *P* = 0.037) and surgical treatment (29.5% vs. 2.9%, *P* < 0.001) were common in type A. However, there were no significant difference in-hospital mortality (4.9% vs. 2.9%, *P* = 0.671) and 2-year mortality (13.1% vs. 11.5%, *P* = 0.765) between type A and B. In the type A patients, there were no significant difference in in-hospital mortality (7.1% of surgery vs. 4.3% of medical, *P* = 0.428) and 2-year mortality (7.1% of surgery vs. 14.9% of medical, *P* = 0.450) in terms of initial treatment strategy (Figure 3).

**Table 4:** Treatment strategy and clinical outcomes according to location of aortic intramural hematoma

	**Type A (n = 61)**	**Type B (n = 104)**	***P *** **value**
**Admission duration (days)**	19 ± 11	17 ± 13	0.535
Emergency surgery (within 24 hrs)	29 ± 14	26	0.855
Medical therapy and timely surgery	16 ± 8	17 ± 13	0.404
**In-hospital mortality**	3/61 (4.9%)	3 /104 (2.9%)	0.671
Emergency surgery (within 24 hrs)	1/14 (7.1%)	0/1 (0%)	1.000
Medical therapy and timely surgery	2/47 (4.3%)	3/103 (2.9%)	0.649
**2-year mortality**	8/61 (13.1%)	12/104 (11.5%)	0.765
Emergency surgery (within 24 hrs)	1/14 (7.1%)	0/1 (0%)	1.000
Medical therapy and timely surgery	7/47 (14.9%)	12/103 (11.7%)	0.580
Progression to aortic dissection for 2 years	11 (18.0%)	7 (6.7%)	0.037
Surgical treatment for 2 years	18 (29.5%)	3 (2.9%)	< 0.001

## Discussion

Aortic IMH, known as a variant form of aortic dissection, is characterized by the absence of intimal tear and direct flow communication between true and false lumen. It results from spontaneous rupture of aortic vasa vasorum or penetrating atherosclerotic ulcer [10-12]. Recent advances in noninvasive imaging such as computed tomography (CT), magnetic resonance (MR) imaging, and transesophageal echocardiography (TEE) are used to identify a variant of aortic dissection and have further emphasized the importance of early diagnosis of acute aortic syndrome. Among them, CT is widely used as the imaging modality of first choice for the assessment of thoracic aortic disease and can be used even in clinically unstable patients. Moreover, various diameters of the aorta can be measured easily in serial CT images such as aortic diameter and aortic wall thickness [1,13-15]. There are several studies that propose these parameters as a prognostic factor. Maximum aortic diameter ≥ 50 mm, hematoma thickness ≥ 11 mm are supposed to predict adverse clinical outcome [16,17]. Our study also showed that patients with larger hematoma thickness (≥10 mm) at initial CT were considered as a high risk group. Perhaps this is because larger hematoma thickness might represent a larger blood accumulation in the aortic wall, which could increase the risk of development of aortic dissection or perivascular leakage.

The management of IMH of the aorta remains controversial. Existing studies have suggested that different therapeutic strategy based on the affected area be used as the site of IMH is an important parameter to determine prognosis. Usually patients with distal IMH involving the descending aorta (Stanford type B) treated medically and their favorable prognosis is demonstrated in many studies. Kaji et al. reported long-term clinical course of patients with type B IMH. In their study, treated initially with medical therapy, type B IMH shows lower in-hospital mortality (0% vs. 14%, *P* = 0.006) and higher 5-year survival rates (97% vs. 79%, *P* = 0.009) compared with type B aortic dissection [18]. Song et al. reported that absence of persistent flow communication in the false lumen resulted in a favorable remodeling process in IMH affecting distal descending aorta [19]. It has been reported that even type B IMH could progress to overt dissection or aortic rupture [20]. Medical therapy is generally accepted in initial management of type B IMH.

However, management of patients with type A IMH is controversial. Some studies reported that medically treated type A IMH patients showed worse prognosis than surgically treated patients. Because short term prognosis is serious in IMH involving the ascending aorta owing to frequent progression to aortic rupture, dissection or aneurysm, they insist that surgical repair improves outcome in type A patients [7,21]. However, the progression of type A IMH appears to be more benign than that of aortic dissection and studies from Asian countries reported low mortality rates in medically treated proximal IMH. Song et al. reported that among medically treated type A IMH patients, 67% showed disappearance of hematoma and 78% survived during 3-year follow-up [9,22]. In another study of type A patients treated initially with supportive medical therapy, in-hospital mortality rate was significantly lower (7% vs. 34%, *P* = 0.004) and 5-year survival rates was significantly higher (90% vs. 62%, P = 0.004) than aortic dissection group [23]. Another recent study conducted for North American and European showed that the majority of type A IMH patients (84.4%) received surgical treatment. Even though there was no direct comparison between surgically treated and medically treated type A IMH, the mortality in medically treated group (40.0%) was much higher than that of surgically treated group (24.1%) [24]. Although the exact cause is unknown, IMH is diagnosed much more frequently and has better outcomes in Japan/Korea than North America/Europe. It is possibly due to the heightened awareness of the IMH diagnosis and detect more benign variant of IMH. In addition, genetic, dietary, or environmental factors may influence geographic variation [25].

Previous studies have shown that medical treatment alone was not enough to manage all type A IMH patients. Patients with recurrent pain, progression to overt type A dissection, or progressive aortic dilatation during medical follow-up should converted to surgery [26]. To monitor the morphological changes and development of delayed vascular complications such as that, regular follow-up imaging will be essential [27]. In addition, recent technical improvements in surgery for acute aortic syndrome have resulted in reduced operative mortality. Moreover, delayed surgery did not increase mortality [8,28]. Thus, supportive medical treatment with frequent follow-up imaging studies and timed surgical repair can be a rational therapeutic strategy in management of type A IMH. In this study, we analyzed 165 Asian patients with IMH, and this is one of the largest studies of IMH to date. There were also no significant difference in in-hospital mortality and 2-year mortality according to initial treatment strategy. Based on our data, medical therapy with or without timely operation resulted in favorable long-term results similar to other studies from Asia.

There are several limitations in our study. First, it was a retrospective analysis of data and not a randomized study. There were no definite criteria for surgical therapy. Second, the rates of CT follow-up were low and this may underestimate progression of IMH. Third, there were a relatively small number of patients who developed aortic dissections or who died. Thus, we did not have adequate power to identify risk factors of these outcomes. Fourth, we did not take into account the variations of normal aortic dimensions related to age.

## Conclusions

For real world practice in Korea, most of IMH patients were treated medically at presentation and showed favorable outcomes. Thus, even in type A acute IMH, early medical treatment with alternative surgical conversion for selected, complicated cases would be a favorable treatment option. Moreover it can also reduce medical costs through the shorter hospitalization period and the reduction of operation charge. Longer follow-up observation of more patients is needed to assess the real impact of the initial hematoma thickness on the prognosis of IMH and timing of surgical intervention.

## Abbreviations

IMH: Intramural hematoma of the aorta; CT: Computed tomographic; MR: Magnetic resonance; TEE: Transesophageal echocardiography.

## Competing interests

The authors declared that they have no competing interests.

## Authors’ contributions

YJC prepared all the data, acquisition of data, analysis and writing the draft, JWS participated in the data collection, analysis and interpretation of data, SHL participated in the data collection and discussion, UK participated in the data collection and discussion, DGS participated in the data collection, analysis and interpretation of data and revision, YJK participated in the data collection and supervised the acquisition of data, SHH participated in the data collection, CWN participated in the data collection, YKC participated in the data collection, BRL participated in the data collection, BCJ participated in the data collection, JBL participated in the data collection, JKR participated in the data collection, HSP participated in the data collection, JHL participated in the data collection, SYJ participated in the data collection and interpretation of CT images, JSP initiated the study and supervised the acquisition of data, helped the final approval of the version to be published and wrapped up the manuscript. All authors read and approved the final manuscript.

## Pre-publication history

The pre-publication history for this paper can be accessed here:

http://www.biomedcentral.com/1471-2261/14/103/prepub
